# Risk prediction models for enteral nutrition feeding intolerance in critically ill patients: an overview of systematic reviews

**DOI:** 10.3389/fnut.2025.1662409

**Published:** 2025-10-01

**Authors:** Zhenfeng Zhou, Jicheng Zhang, Chunmei Fan, Zhengang Wei, Qi Wang, Congcong Liu

**Affiliations:** Critical Care Medicine Ward II, Shandong Provincial Hospital Affiliated to Shandong First Medical University, Jinan, China

**Keywords:** enteral nutrition, feeding intolerance, risk prediction model, systematic review, meta-analysis, critical care

## Abstract

**Objective:**

To evaluate the systematic reviews of a risk prediction model for enteral nutrition feeding intolerance in critically ill patients.

**Methods:**

We registered the protocol for this overview in PROSPERO. Computer searches were conducted on the PubMed, Embase, Web of Science, Cochrane Library, CINAHL, Embase databases China National Knowledge Infrastructure (CNKI), Wanfang Database and SinoMed to search for systematic reviews related to the study. Two investigators independently screened the literature, extracted information, and used the Risk of Bias in Systematic Reviews (ROBIS) tool to assess the risk of bias of the retrieved systematic reviews.

**Results:**

Eight systematic reviews were included, total of 115 prediction models, with more than half of the predictive models (71/115 = 61.7%) having undergone internal validation and a small number (36/115 = 31.3%) having undergone external validation. Of the quality evaluations, two were at low risk of bias, six were at high risk, and the overall risk of bias was high.

**Discussion:**

The completeness of reporting and methodological quality of systematic reviews of prediction models for enteral nutrition feeding intolerance in critically ill patients were inconsistent and lacked specific quality standards. There is an urgent need for standardized reporting and quality criteria to improve the quality of prediction models for enteral nutrition feeding intolerance in ICU patients.

## 1 Introduction

In the intensive care unit (ICU), Critically ill patients often require nutritional support due to the severity of their condition, which made them prone to intestinal dysfunction, reduced intake, and immune dysfunction of the body (1). Enteral nutrition (EN), as an important method of nutritional support, is the preferred method of nutritional support for ICU patients recommended by the guidelines because it meets the physiological needs, helps to repair the intestinal mucosal function of patients, enhances the function of the gastrointestinal tract, and improves the immunity of the organism (2, 3). The European Society for Clinical Nutrition and Metabolism (ESPEN) guidelines recommend that enteral nutrition be administered within 48 h of admission to critically ill patients without contraindications to enteral nutrition (4). However, in clinical practice, enteral nutrition feeding intolerance may occur due to high levels of stress in the body, which can damage the gastrointestinal mucosal barrier and lead to metabolic imbalance (5, 6). The medical community defines feeding intolerance (FI) as the development of gastrointestinal symptoms—including vomiting, reflux, diarrhea, constipation, bloating, high gastric residuals, and gastrointestinal hemorrhage—during enteral nutrition. It represents one of the most prevalent gastrointestinal complications in ICU patients receiving enteral nutrition (7, 8). Some research findings indicate that the incidence of FI during enteral nutrition in critically ill patients ranges from 2% to 75% (9). The occurrence of FI not only leads to nutritional disruption but also prolongs the patient's hospital stay, increases the duration of mechanical ventilation, and adds to the patient's medical burden, with a close relationship to poor patient outcomes and prognosis, including secondary infections and death (10, 11). Therefore, early identification of patients at high risk of FI and implementing measures to intervene are crucial for improving the prognosis of critically ill patients.

Accurate assessment of FI in critically ill patients remains challenging, particularly in comatose or deeply sedated patients. It can be evaluated based on the Acute Gastrointestinal Injury (AGI) severity grading proposed by the European Society of Intensive Care Medicine (ESICM) (12), or by measuring gastric residual volume (GRV) to assess FI (13, 14). Lin et al. (15) developed a feeding intolerance assessment scale based on gastrointestinal symptoms. Nevertheless, they fail to comprehensively cover the key factors affecting the nutritional status of critically ill patients, making it difficult to predict clinical outcomes accurately (16). Prediction models are methods for quantitatively estimating the risk of developing a disease or experiencing a future outcome. They achieve this through multifactorial analyses and the combination of multiple predictors (17, 18). The prediction model can identify predictors of FI. To do this, it incorporates general indicators of a patient's current or past history and laboratory findings. It also mines the data for potentially complex associations. Through these steps, the model can more accurately predict the outcome of critically ill patients who may develop FI (19). Therefore, it is necessary to develop prediction models for enteral nutrition feeding intolerance in critically ill patients. Numerous scholars have developed various prediction models for F in critically ill patients (20–22). However, considerable differences remain among the models regarding the applicable population, performance, and clinical applicability, which still require further optimization and research.

With the continuous development of prediction models for critical care enteral nutrition feeding intolerance, researchers have conducted systematic reviews (SRs) and meta-analyses of FI prediction models to comprehensively search for and critically evaluate the accuracy, discriminatory nature, and external validity of these models. Such SRs can help us identify reliable prediction models. For these SRs to draw reliable and transparent conclusions, they must adhere to specific methodological standards (17, 23); however, methodological reporting bias may affect the quality of the SRs. We have not identified any studies evaluating the methodological quality of such SRs, and their level of evidence remains unclear. Therefore, this study aims to provide an overview of the existing systematic reviews of feeding intolerance prediction models for critically ill patients to provide healthcare professionals with more evidence-based clinical decision support for managing critically ill patients with feeding intolerance.

## 2 Materials and methods

We adhered to the Cochrane Handbook and registered this study on the PROSPERO website (registration number CRD420250654359). We based this study on the Preferred Reporting Items for Overviews of Systematic Reviews (24), including the harms checklist (PRIO-harms) (25), a reporting framework that evolved from the PRISMA harms checklist (26). Additionally, we have referred to the key steps summarized in the guidelines published by BMJ Medicine (27).

Our study employed the PICOTS framework to ensure rigorous assessment and data extraction from studies focused on prediction modeling within systematic review contexts (28). This framework facilitates clear articulation of the review's objectives, delineation of search strategy methodologies, and establishment of criteria for study inclusion and exclusion. The following sections detail the key components of our systematic review:

P (Population): critically ill patients;

I (Intervention): a feeding intolerance risk prediction model tailored specifically for patients receiving enteral nutrition;

C (Comparison): no specific control, focusing on prediction models;

O (Outcome): feeding intolerance;

T (Time): during ICU stay;

S (Setting): intensive care unit.

### 2.1 Inclusion and exclusion criteria

Inclusion criteria: the study subjects were adult ICU patients (age ≥18 years) undergoing enteral nutrition; the type of study was systematic reviews and Meta-analysis; the study was the construction and/or validation study of feeding intolerance prediction model for enteral nutrition in critically ill patients; The languages were Chinese and English. Exclusion criteria: systematic reviews proposals, traditional reviews, conference abstracts, etc.; literature for which the full text is unavailable; and duplication of published literature.

### 2.2 Search strategy

We searched PubMed, Web of Science, Cochrane Library, CINAHL, Embase databases, the China National Knowledge Infrastructure (CNKI), Wanfang Database and SinoMed. We conducted the search using the following terms: critical illness/intensive care/intensive care units/critical^*^/ICU, feeding intolerance/feed^*^ intolerance/intolerance, prediction model/predict^*^/risk^*^/model^*^/risk prediction/model/risk calculation/risk score/AUC/ROC curve/c statistic/validat^*^/decision^*^/clinical^*^. Researchers conducted the search using subject terms and free words and further searched the references incorporated into the literature. The search is open from the build date to August 25, 2025. Taking the PubMed database as an example, the retrieval formula can be found in Supplementary material 1.

### 2.3 Screening

We used the literature management software EndNote X9. Two researchers independently screened and cross-checked the literature according to the inclusion and exclusion criteria. Read the titles and abstracts to exclude irrelevant literature, and then obtain further full text for detailed independent screening. If there were disagreements, they would be negotiated and resolved through a third researcher. We screened the literature using the PRISMA 2020 literature screening process (29).

### 2.4 Data extraction

Two researchers were responsible for extracting the data. In the event of disagreement, we consult a third party to assist in making a judgment. Use pre-designed data extraction forms to ensure the accuracy and completeness of data extraction. Extracted content mainly includes first author, year of publication, country of the first author, number of included literature, number of predictive models, number of patients, modeling method, validation method, and risk of bias evaluation tool. Contact the review authors to identify any missing information or inconsistencies in the reported data, if necessary.

### 2.5 Data synthesis and analysis

The included literature was read and analyzed repeatedly by two researchers. The extracted data were presented in tabular form to summarize and descriptively analyze the findings. The systematic review incorporates primary studies with variations in study populations, modeling methodologies, and model performance metrics, which results in substantial heterogeneity. The included systematic reviews (SRs) may or may not have conducted meaningful statistical meta-analyses. Therefore, we have not performed quantitative analyses (meta-analysis/subgroup/meta-regression analysis), only descriptive analyses.

### 2.6 Risk of bias assessment

Evaluation of the risk of bias of included studies according to the Risk of Bias in Systematic Reviews (ROBIS) tool (30). Two independent researchers trained in proficiency performed the evaluation and cross-checking. In case of disagreement, a third researcher made the judgment. The ROBIS tool consists of three phases. Phase 1 was an optional assessment of relevance, which was not undertaken in this overview. Phase 2 was aimed to identify biases in the review process and included four areas: (A) study eligibility criteria, (B) identification and study selection, (C) data collection and study evaluation, and (D) synthesis and discovery. The concern for bias associated with each domain was assessed as “low,” “high,” or “unclear.” Phase 3 aimed to assess the overall risk of bias by summarizing the findings of Phase 2. Investigators judged the overall risk of bias as “low,” “high,” or “unclear.”

## 3 Results

### 3.1 Search results

**Figure 1:**
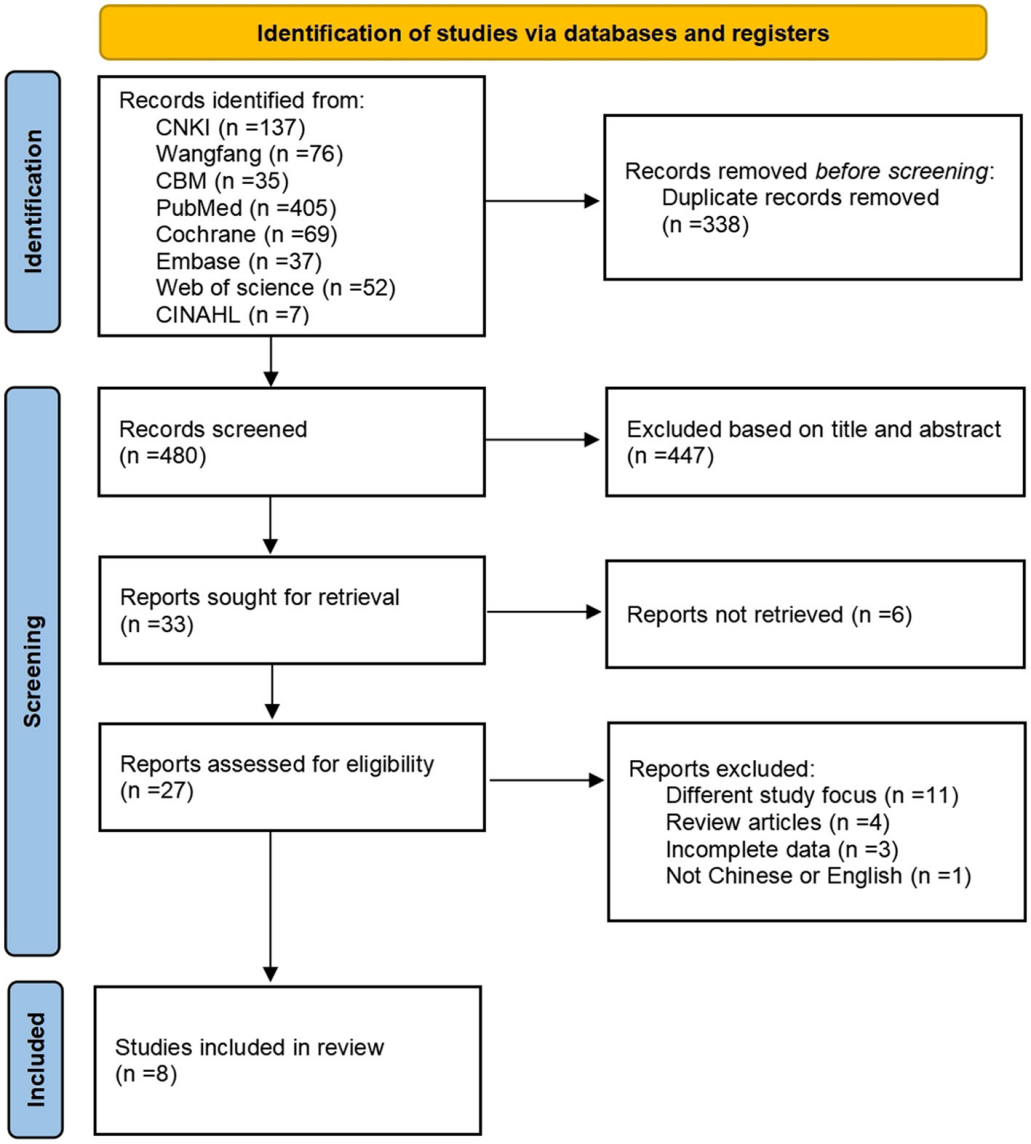
PRISMA 2020 literature screening flowchart.

We retrieved 818 articles for this study, and 480 remained after deleting duplicates; 27 remained after two researchers screened the articles by title and abstract, removing those articles for which full text was unavailable. We excluded 19 articles that did not meet the inclusion and exclusion criteria. Ultimately, we included eight systematic reviews in this study. Figure 1 shows the PRISMA 2020 literature screening process.

### 3.2 Characteristics of included studies

The SRs included in this study all involved retrospective and prospective studies. The first authors of the SRs are all from China. The included SRs synthesized literature published from 2013 to 2024. Each SR included 9–19 literature studies, with a total of 94 literature studies. The sample size ranged from 1,635 to 4,478 individuals, and the study population consisted of patients from comprehensive ICUs, neurologic ICUs, emergency ICUs, geriatric ICUs, those with severe pancreatitis, and those with sepsis. The number of prediction models ranged from 9 to 24, totaling 115. Most models used logistic regression, while others used machine learning approaches. More than half of these prediction models were internally validated (71/115 = 61.7%), and only (36/115 = 31.3%) were externally validated. All SRs used the prediction model risk of bias assessment tool (PROBAST) to evaluate the risk of bias in the prediction models. Table 1 summarizes the characteristics of the included studies.

**Table 1:** Characteristics of included systematic reviews.

**First author, year**	**Country**	**Included participants**	**Included studies/model**	**Internal validation reports**	**External validation report**	**Model development method**	**Risk of bias tool**
Yang (2024) (35)	China	2,394	9/13	7	4		PROBAST
Li (2024) (36)	China	1,635	9/9	5	3		PROBAST
Chen (2024) (37)	China	1977	10/14	8	1		PROBAST
Chen (2025) (34)	China	3,527	14/18	14	7		PROBAST
Wang (2024) (55)	China	3,392	10/10	6	3		PROBAST
Yang (2024) (38)	China	4,478	19/24	10	6		PROBAST
Liu (2023) (39)	China	2,643	10/14	13	7		PROBAST
Huang (2025) (50)	China	2,791	13/13	8	5		PROBAST

LR, Logistic regression; DL, Deep learning; artificial neural network; ML, machine learning; RF, random forest; NB, Naive Bayes; GBT, gradient boosted tree; factor analysis; Nomogram; PROBAST, prediction model risk of bias assessment tool.

### 3.3 Risk of bias assessment of systematic reviews

**Figure 2:**
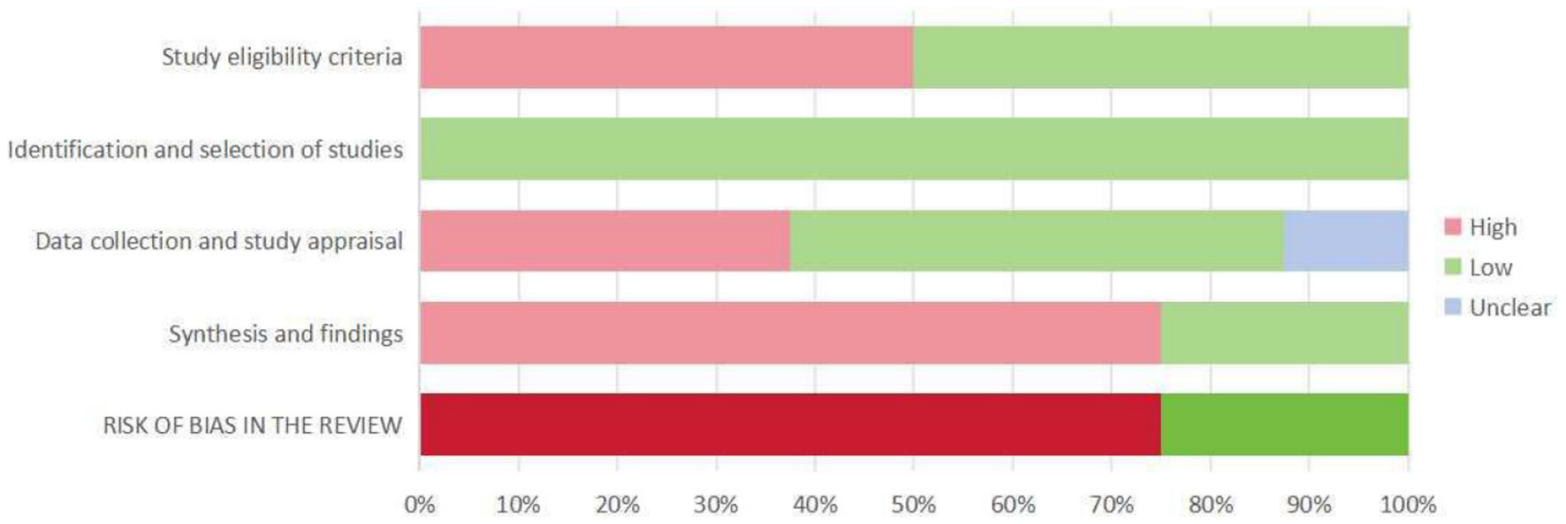
Summary of ROBIS assessment. Percentages derived from the number of included systematic reviews (100% = 8 included systematic reviews).

Figure 2 illustrates the assessed risk of bias for each domain and the overall assessment as a percentage of the included SRs. Table 2 contains individual ratings for the signaling issues included in each domain. We rated two systematic reviews as having a low risk of bias and six as having a high risk of bias. The study found that there was a relatively high risk of bias in the included systematic reviews. Although the process of identifying and selecting studies was generally robust, other domains, such as study eligibility criteria, data collection and appraisal, and especially synthesis and findings, exhibited considerable proportions of high bias risk, contributing to the elevated overall risk of bias. The high risk of bias was mainly due to the lack of reference to pre-designed protocols, the absence of accessible versions, and the absence of reference to or integration of data.

**Table 2:** Summary of ROBIS assessment.

	**Phase 2**																					**Phase 3**
**First author, year**	**1 Study eligibility criteria**					**2 Identification and selection of studies**					**3 Data collection and study appraisal**					**4 Synthesis and findings**						**Risk of bias**
	**1.1**	**1.2**	**1.3**	**1.4**	**1.5**	**2.1**	**2.2**	**2.3**	**2.4**	**2.5**	**3.1**	**3.2**	**3.3**	**3.4**	**3.5**	**4.1**	**4.2**	**4.3**	**4.4**	**4.5**	**4.6**	
Yang (2024)	N	PY	PY	PY	Y	PY	NI	Y	Y	Y	PY	PY	PN	Y	Y	PY	NI	N	N	N	N	High
	High					Low					Unclear					High					
Li (2024)	N	PY	PY	PY	Y	PY	Y	Y	Y	Y	PY	PY	N	Y	Y	PY	NI	N	N	N	N	High
	High					Low					High					High						
Chen (2024)	Y	Y	Y	PY	Y	PY	Y	Y	Y	Y	Y	Y	Y	Y	Y	Y	PY	PY	Y	Y	Y	Low
	Low					Low					Low					Low						
Chen (2025)	Y	PY	PY	Y	Y	PY	Y	Y	Y	Y	Y	Y	Y	Y	Y	Y	Y	PY	Y	Y	Y	Low
	Low					Low					Low					Low						
Wang (2024)	N	Y	PY	PY	Y	PY	Y	Y	Y	Y	Y	Y	N	Y	Y	PY	Y	NI	NI	N	N	High
	High					Low					High					High						
Yang (2024)	Y	PY	PY	Y	Y	PY	PY	Y	Y	Y	Y	Y	N	Y	Y	PY	NI	PN	N	N	N	High
	Low					Low					Low					High						
Liu (2023)	Y	PY	PY	Y	Y	PY	PY	Y	Y	Y	PY	PY	Y	Y	Y	PY	NI	PN	N	N	N	High
	Low					Low					Low					High						
Huang (2025)	N	PY	Y	Y	Y	PY	PY	Y	Y	Y	Y	Y	Y	Y	Y	PY	PY	NI	N	N	High
	High					Low					Low					High						

Y, yes; PY, probably yes; NI, no information; PN, probably no; N, No.

## 4 Discussion

Enteral nutrition feeding intolerance in critically ill patients has become a key clinical problem in critical care nutrition support. Traditional static nutritional assessment tools fail to capture dynamic changes in critically ill patients (31). In contrast, prediction models demonstrate the potential to accurately predict FI by integrating multidimensional data, such as physiologic indicators and laboratory parameters (32). However, not every prediction model is suitable for the clinical environment. It is a challenging task for clinicians to independently assess the reliability of each predictive model and select the most appropriate risk prediction models. Evidence-based medicine guidelines regard systematic reviews as the highest level of evidence, and SRs of prediction models are important because they enable a thorough assessment of existing models using specific tools to identify those with reliable and accurate results (33). In this study, we provide an overview of the systematic reviews of FI prediction models for enteral nutrition in critically ill patients and critically assess the methodological quality and reporting standards of existing SRs.

This study included a total of 8 SRs with 115 models from comprehensive ICUs, neuro-ICUs, patients with severe pancreatitis, and those with sepsis. The methods of constructing prediction models included Nomogram, Logistic Regression, Random Forest, and Deep Learning methods. One report (34) included a prediction model with some methodological flaws in the development and validation process. The main reasons can be reflected in the following aspects. First, predictors are included in the definition of endpoints. Second, there is reliance on retrospective data sources. Third, the sample sizes are small. Fourth, continuous variables and missing data are handled improperly. Fifth, the assessment of model performance is incomplete. These reasons caused most prediction models to receive a high risk of bias rating and low applicability score in PROBAST. Other studies have reported similar results (35–38). Some studies (36, 39) included only a few dozen patients, making it difficult to fully represent the characteristics and conditions of different patient types. Some studies (21, 40) had missing or inaccurate data during the data collection process, which can affect the accuracy and reliability of the model.

In addition, our study found that 61.7% of the models were internally validated, while only 31.3% were externally validated. Furthermore, the external validation cohorts were predominantly from a single center or a homogeneous population. A contradiction exists between the high percentage of internal validation and the lack of external validation. This contradiction reveals characteristics of the model. On one hand, the model exists and performs well in the original data. On the other hand, it lacks generalizability across populations (41). Therefore, in clinical practice, the predictive performance of the models may be lower than the reported results, leading to unreliable predictions. We recommend that prospective studies be incorporated whenever feasible in the development of predictive models, accompanied by rigorous external validation.

Each stage of the systematic review production process has the potential for bias, and researchers must consider these potential biases when interpreting the results and conclusions of the SRs (42, 43). Therefore, the risk of bias in SRs must be evaluated using specific evaluation tools. There are no specialized tools for evaluating the methodological quality of prediction models' SRs, and there is a lack of clear criteria for assessing the risk of bias (44).

Although AMSTAR 2 (A Measurement Tool to Assess Systematic Reviews tool of Version 2) is rigorously formulated, widely used, and actionable, its scope of adaptation does not include diagnostic test systematic reviews, network Meta-analyses, single-case data meta-analyses, and profile evaluations (45, 46). This study used the ROBIS tool (30, 47), which can assess the risk of bias for various SRs, including interventional, diagnostic, etiologic, prognostic, and others.

We assessed six of the eight included SRs as having a high risk of bias and two as having a low risk. The higher risk of bias stemmed from the lack of a clear statement that the review methodology was established prior to the review and that it was not registered in platforms such as the Cochrane Library and PROSPERO. Systematic reviews are observational studies, and the methodology should be agreed upon before the review begins. Following a well-established protocol reduces the risk of review bias, avoids duplication of studies, and ensures transparency (48). On the other hand, it primarily focuses on the fact that some studies do not mention or fail to integrate data. This result may be because the original studies included in the SRs had subjects from different ICUs and patients with various disease types, and the methods used to construct the prediction models varied, resulting in greater heterogeneity. In addition, the original study had data quality issues, including insufficient sample size, non-reporting of missing data, and treatment methods that only used the deletion method to handle missing data, which is likely to result in excessive loss of sample size and significant errors. Therefore, we recommend expanding the sample size, identify the causes of missing data, and employ methods such as imputation and different modeling approaches to handle this issue (49).

However, studies (50, 51) have pointed out that the study heterogeneity is too high, such as significant differences in the sources of research participants and the difficulty of unifying the construction methods of prediction models. Alternatively, only qualitative descriptions of different prediction models, comparison of characteristics and application scenes, rather than quantitative analysis of model performance. Forcing data integration may bias results, making data integration inapplicable in such cases (52). The quality of a prediction model determines the quality of its systematic review. Therefore, we recommend strictly adhering to methodological standards such as the TRIPOD statement (53) when developing predictive models to ensure adequate reporting and methodological quality. Additionally, systematic reviews of prediction models should be conducted in strict compliance with these standards (23, 54). Meanwhile, researchers need to develop quality evaluation tools suitable for SRs of prediction models to help evaluate their quality and risk of bias more accurately.

## 5 Limitations and outlook

Although this study conducted a detailed literature search, and two researchers independently assessed and cross-checked the data during literature screening, data extraction, and analysis, as well as the risk of bias assessment, we still found several problems. In the literature search, we included only Chinese and English literature, and omission of gray literature searches, which may have missed other high-quality studies. Moreover, the first authors of the included SRs were from China, which may have resulted in geographic bias that could have affected the completeness of the evidence. In addition, our study focused only on the methodological evaluation level of the SRs and did not directly assess the clinical effectiveness of the original prediction models. In future research, researchers should improve the performance and quality of prediction models. Expanding the scope of research to focus on different types of patient groups, conducting in-depth exploration of the pathogenesis of enteral nutrition feeding intolerance, optimize predictors and models, and constructing more accurate and practical risk prediction models. In terms of methodology, researchers should strengthen the rigor of the study design to ensure the accuracy and completeness of the sample data, thereby improving the integrity and transparency of the study.

## 6 Conclusion

This study comprehensively evaluated the FI prediction models for critically ill patients through an overview of SRs. Research on FI prediction models for critically ill patients is still in the developmental stage, with an imperfect model construction system and variable quality. The deficiencies in methodological quality and reporting specifications of SRs' prediction models, as well as the lack of high-quality SRs, highlight the urgency of establishing harmonized reporting standards, strengthening multicenter external validation, and developing dedicated quality assessment tools. To improve the quality of prediction models for enteral nutrition FI in critically ill ICU patients and to provide more reliable evidence to support early and precise intervention in feeding intolerance.
