# Supplementary material for: Risk prediction models for enteral nutrition feeding intolerance in critically ill patients: an overview of systematic reviews
Source: Front Nutr. 2025 Oct 1;12:1662409. doi: 10.3389/fnut.2025.1662409 (PMC12520958; doi:10.3389/fnut.2025.1662409)
Supplement: Supplementary file 1 [file Table_1.docx]

Supplementary Material 1

# Retrieval formula

Taking the PubMed database as an example, the retrieval formula is as follows:

("critical illness" [Mesh] OR "intensive care" [Mesh] OR "intensive care units" [Mesh] OR critical*[All Fields] OR intensive*[All Fields] OR ICU [All Fields]) AND ("feeding intolerance" [Mesh] OR feed* intolerance [All Fields] OR intolerance [All Fields]) AND ("prediction model" [Mesh] OR predict*[All Fields] OR risk*[All Fields] OR model*[All Fields] OR "risk prediction" [All Fields] OR model [All Fields] OR "risk calculation" [All Fields] OR "risk score" [All Fields] OR AUC [All Fields] OR "ROC curve" [All Fields] OR "C statistic" [All Fields] OR validat*[All Fields] OR decision*[All Fields] OR clinical*[All Fields]).
